# Prevalence and Risk Factors for Dental Caries among Preschool Children: A Cross-sectional Study in Eastern India

**DOI:** 10.5005/jp-journals-10005-1518

**Published:** 2018-06-01

**Authors:** Vinay K Chugh, Kushal K Sahu, Ankita Chugh

**Affiliations:** 1 Research Assistant, Department of Dentistry, All India Institute of Medical Sciences Jodhpur, Rajasthan, India; 2 Associate Professor, Department of Dentistry, All India Institute of Medical Sciences Jodhpur, Rajasthan, India; 3 Associate Professor, Department of Dentistry, All India Institute of Medical Sciences Jodhpur, Rajasthan, India

**Keywords:** Breastfeeding, Dental caries, Oral hygiene behavior, Preschool children.

## Abstract

**Introduction:**

Dental caries is one of the major widespread health issues that continue to negatively affect the oral health of children globally.

**Aim:**

To estimate the prevalence of dental caries and its risk factors among preschool children of Bhubaneswar, Odisha, India.

**Materials and methods:**

The study was a community-based cross-sectional one among preschool children with (N = 425) participants recruited from the Anganwadi centers (AWCs) of Bhubaneswar, Odisha, India. By using a cross-sectional study design, dental caries was assessed using the World Health Organization (WHO) guidelines, and other socioeconomic and risk factors data were collected through parental interview using questionnaires. Caries was identified at both tooth and surface levels through visual dental examinations by trained and calibrated dentists. Logistic regression analyses were used to identify associations among variables and caries.

**Results:**

The proportion of preschool children suffering from dental caries was found to be 47.29%. The multivariable-adjusted model depicted that longer duration of breastfeeding was a significant predictor as follows: Those who had breastfed for more than 36 months [adjusted odds ratio (AOR): 5.41; 95% confidence interval (CI): 2.97-9.85; p = 0.001], 12 to 24 months (AOR: 2.1; 95% CI: 1.04-4.36; p = 0.037), followed by increase in age 61 to 72 months (AOR: 5.39; 95% CI: 2.72-10.67; p = 0.001), 49 to 60 months (AOR: 2.53; 95% CI: 1.41-4.52; p = 0.002), more than two children in the family (AOR: 2.70; 95% CI: 1.55-4.69; p = 0.001), and children who did not brush the teeth under the parent’s supervision (AOR: 2.70; 95% CI: 1.55-4.69; p = 0.001).

**Conclusion:**

The study highlights the need to increase awareness about the oral health and hygiene among parents of preschool children in India.

**How to cite this article:** Chugh VK, Sahu KK, Chugh A. Prevalence and Risk Factors for Dental Caries among Preschool Children: A Cross-sectional Study in Eastern India. Int J Clin Pediatr Dent 2018;11(3):238-243.

## INTRODUCTION

Dental caries is one of the major widespread health issues that continues to negatively affect the oral health of children globally.^1^ The prevalence of dental caries varies across the different provinces in the USA 11 to 53%, in Sri Lanka 32.1%, and in India 44%.^2^ In addition, the occurrence of dental caries in South Africa was found to be 23.3%,^3^ and in Pakistan 51%.^4^ According to the WHO, dental caries is defined as “the localized, post-eruptive, pathological process of external origin involving softening of the hard tooth tissue and proceeding to the formation of a cavity.” It is a hastily succeeding disease among preschool children which involves the primary maxillary anterior teeth and posterior teeth, whereas the mandibular anterior teeth are less affected because of the rapid saliva formation which helps to wash out the ingredients required for bacterial growth.^5^ Dental caries commonly involves more than one aspect for causing the dental caries, such as the host (teeth), the substrate (sugary content food), bacteria, and time.^6^ Principally, the bacteria and sugary food together act to form an acid production that results in the formation of teeth cavitation.^7^ The bacteria which are mainly responsible for causing the dental caries are *Streptococcus mutans, Lactobacillus,* and other bacteria that are also linked, such as *Veillonella* spp, *Actinomyces* spp, and *Bifidobacterium* spp.^8^ The risk factors responsible for early childhood caries, such as the low socioeconomic status, poor underweight children,^9^ breastfeeding,^10^ changing lifestyle, and the poor dietary pattern of the parents^11^ are all considered to be the predictors of dental caries. The burden of dental caries is increasing day by day due to the changing lifestyle and dietary pattern of the parents. Undoubtedly, dental caries is a major oral health problem; if it is left untreated, it can lead to the decayed tooth, severe pain, which affect the growth and maturation of secondary dentition (permanent dentition) which leads to malocclusion of teeth. There is also the possibility of compromised chewing habit and it can compromise the weight gain of the child. The situation can be prohibited from having the thorough knowledge about the feeding and oral hygiene practices. Since no studies are available as per our knowledge regarding the prevalence and its risk factors associated with dental caries among the preschool children in Bhubaneswar, Odisha, India the aim of the study is to estimate the prevalence and its risk factors associated with dental caries among preschool children in Bhubaneswar, Odisha, India.

## MATERIALS AND METHODS

This study was conducted using a cross-sectional plan based on the preschool children in Bhubaneswar, Odisha, India readily regarded as the temple city of India. It contains nearly about 213,000 under 6 years age, out of which in urban and rural areas, there are 81,000 and 131,000 respectively.^12^ The ethical authorization for the study was taken from the Institutional Ethical Committee of Asian Institute of Public Health along with the permission from the local authority, and informed consent was taken individually from the study participants. The target population of our study consisted of children between 3 and 6 years of age and their mothers. Inclusion criteria: Children between 3 and 6 years of age who were enrolled in AWCs of Bhubaneswar, Odisha, India and their parents who had signed the informed consent. Exclusion criteria: Children who were absent from the AWCs at the time of screening and their parents who had not given the informed consent. The sample size after calculation was found to be 380, but keeping the probability of participants’ absence from the AWCs, the sample size was increased by 11% and the final sample size was set at 425. A total of 425 participants were calculated with the help of OpenEpi sample size calculator based on the prevalence 44%^2^ along with 95% CI, 5% precision, and power 80%. The random type of sampling method was used for recruiting the 425 participants. A total of 35 AWC were selected from the Bhubaneswar randomly along with their participant’s household members. The information about the sociodemographic, socioeconomic factors of the household, feeding, and oral hygiene practices of the parents was obtained using a structured questionnaire. For probing the preschool children, WHO probe and mouth mirror were used for diagnosing dental caries. The WHO guidelines 2013 were followed. The examination was carried out in direct sunlight under the presence of their parents and for sterilization of the instruments, disinfectant solutions and a pressure cooker were used. The examination was carried out under the supervision of an experienced dentist. Prior to this examination, calibration was done; kappa test was used for 26 participants. The mutual agreement between the two examiners was found to be 0.90. The predictor variables for this study are as follows: Age, gender, religion, caste, number of children in a family, socioeconomic status, longer duration of breastfeeding, and brushing under supervision. The data were entered with the help of EpiInfo version 7.2.1.0 for data cleaning, and analysis of the data was done using the STATA version 11.0. Descriptive type of statistics was used for analyzing the estimation of the variables, and analytical statistics were used to find out the risk factors associated with dental caries among preschool children along with the help of a multivariable logistic regression model which was used for adjusting the probable confounders. To present these conclusion, 95% CI, odds ratio (OR), and a p-value<0.05 were considered for significance of the study.

## RESULTS

**Graph 1:**
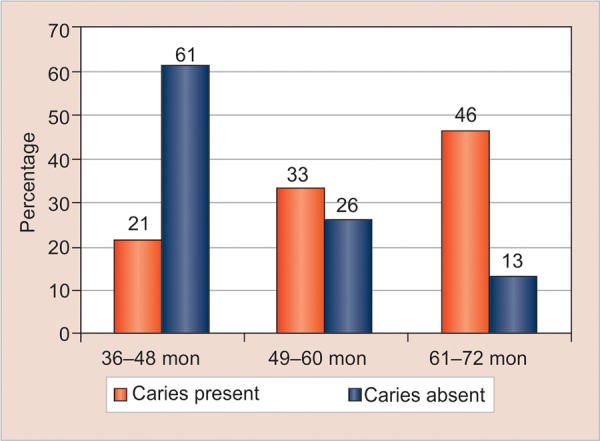
Age-wise distribution of dental caries among preschool children (N = 425)

Table 1 gives information about the sociodemographic profile of the participants. Out of 425 preschool children, 211 (49.65%) belonged to rural settings and 214 (50.35%) were from urban slums. Of the 425 participants examined, 201 (47.30%) were male and 224 (52.70%) were female; 180 (42.35%) were between 36 and 48 months, 124 (29.18%) were between 49 and 60 months, and 121 (28.47%) were between 61 and 72 months. A majority of the participants had two children, 194 (45.65%), followed by 126 (29.65%) who had three children, and 105 (24.70%) had one child. Since the study was carried out in the AWCs of urban slum and the rural areas, it was difficult to capture the higher socioeconomic class which consisted of only 15 (3.53%) of the participants, whereas the lower and middle class had higher percentages, 259 (60.94%) and 151 (35.53%) respectively. Table 2 shows that a total of 425 study participants were examined for dental caries among the preschool children in Bhubaneswar, Odisha, India. Out of 425, 224 (52.71%) were caries-free, whereas 201 (47.29%) had dental caries. Graph 1 states that among the study participants, higher age group 61 to 72 months have greater prevalence (46%) of caries as compared with the younger age group 36 to 48 months (21%), and 49 to 60 months (33%) respectively. Table 3 depicts the relationship between dental caries among preschool children and its associated risk factors, such as age, gender, socioeconomic status, child’s birth order in a family, feeding, and oral hygiene practices of the mother or caretakers. The proportion of preschool children with dental caries increased significantly and was associated with the increase in age (p = 0.001), increased number of children (p = 0.001), and middle and low socioeconomic status (p = 0.001). It also showed that the increased duration of breastfeeding (p = 0.001) was significantly associated with dental caries among preschool children. Also, more children who did not brush the teeth under supervision (p = 0.001) were found to be associated with dental caries. However, no association was found between dental caries with the gender of the children (p = 0.40), caste (p = 0.92), and religion (p = 0.27). Table 4 shows the logistic regression model determining the risk factors for dental caries among preschool children. The model had been adjusted for gender, caste, religion, and socioeconomic status. The multivariable-adjusted model depicted that a longer duration of breastfeeding was a significant predictor for those who had breastfed for more than 36 months (AOR: 5.41; 95% CI: 2.97-9.85; p = 0.001), 12 to 24 months (AOR: 2.1; 95% CI: 1.04—4.36; p = 0.037), followed by increase in age 61 to 72 months (AOR: 5.39; 95% CI: 2.72-10.67; p = 0.001), 49 to 60 months (AOR: 2.53; 95% CI: 1.41—4.52; p = 0.002), more than two children in the family (AOR: 2.70; 95% CI: 1.55^.69; p = 0.001), and children who did not brush the teeth under the parent’s supervision (AOR: 2.70; 95% CI: 1.55-4.69; p = 0.001).

**Table 1:** **Table 1:** Sociodemographic characteristics of the participants (N = 425)

*Variables*		*n*		*%*	
*Place of living*					
Rural		214		49.65	
Urban slums		211		50.35	
*Gender*					
Male		201		47.29	
Female		224		52.71	
*Age of the children (months)*					
36-48		180		42.35	
49-60		124		29.18	
61-72		121		28.47	
*Child’s birth order in a family*					
1st		105		24.70	
2nd		194		45.65	
3rd and above		126		29.65	
*Birth weight of the children (gm)*					
<2,500		81		19.06	
>2,500		344		80.94	
*Religion*					
Hindu		358		84.23	
Muslim		49		11.53	
Christian		18		4.24	
*Caste*					
General		160		37.65	
OBC		140		32.94	
SC		81		19.06	
ST		44		10.35	
*Father’s education*					
No formal education		50		11.77	
Primary school (class up to 7)		110		25.88	
High school (class up to 10)		178		41.88	
College or higher		87		20.47	
*Mother’s education*					
No formal education		68		16.00	
Primary school (class up to 7)		143		33.65	
High school (class up 8 to 10)		178		40.70	
College or higher		41		9.65	
*Occupation of the mother*					
Service		10		2.35	
Business		4		0.94	
Agricultural labor		8		1.88	
Factory/construction labor		19		4.47	
Housewife		384		90.36	
*Occupation of the father*					
Service		61		14.35	
Business		85		20.00	
Factory/construction labor		146		34.35	
Driving		51		12.01	
Agriculture		82		19.29	
*Socioeconomic class*					
Lower class		259		60.94	
Middle class		151		35.53	
Upper class		15		3.53	

## DISCUSSION

Dental caries is usually considered as the most widespread persistent dental predicament that occurs during the early juncture of existence.^13^ Concern and consideration are crucial to avert dental caries among the preschool children. The four risk factors that were found to be concurrent with dental caries among preschool children are: The longer duration of breastfeeding followed by the increasing age of the child, a mounting number of children in a family, and children who did not brush the teeth under the parent’s supervision.

### Prevalence and Dental Caries

The present study shows that the proportion of preschool children suffering from dental caries was found to be 47.29%. A similar type of proportion was found in Kanpur 48%,^14^ Marathahalli 40%,^15^ and Wardha district of Maharashtra 31.81%.^16^ On average, the occurrence of dental caries among preschool children in India was 44%.^2^ In contrast to other countries, the number of dental caries in South Africa was 23.3%,^3^ whereas in Uganda and Tanzania, it was found to be very low, 3.7, and 17.6% in Kampala.^17^ In Kosovo, which is a European country, the commonness of dental caries among preschool children was 17.36%.^18^ In Sudan, the dominance of dental caries was found to be high, 64.6%.^19^ In Asian counties, such as Sri Lanka and Pakistan, the distribution of dental caries was found to be 38.18^7^ and 51%^4^ respectively. The prevalence of dental caries among preschool children shows a similar trend to ours in our neighboring countries, as it was mainly due to sharing of similar kind of intellectual behavior, nutritional model, and socioeconomic category of the people,^20^ whereas in rapidly growing countries, the prevalence was low as compared with budding and underdeveloped countries which was mainly due to increasing awareness about the oral hygiene practices, importance of fluoridation for prevention of dental caries, effective dental awareness given to the people, and parental concern about the dental health.^21^

**Table 2:** **Table 2:** Prevalence of dental caries among preschool children (N = 425)

		*Present*				*Absent*			
*Characteristics*		*n*		*%*		*n*		*%*	
Dental caries		201		47.29		224		52.71	

**Table 3:** **Table 3:** Association between dental caries among preschool children and sociodemographic characteristics, breastfeeding, and oral hygiene practice (N = 425)

		*Present*				*Absent*					
*Characteristics*		*n*		*%*		*n*		*%*		*p-value*	
*Age (months)*											
36-48		43		21.39		137		61.16		0.001*	
49-60		66		32.84		58		25.89			
61-72		92		45.77		29		12.95			
*Gender*											
Male		100		49.75		101		45.09		0.40	
Female		101		50.25		123		54.91			
*Child’s birth order in a family*											
1st		27		13.43		78		34.82		0.001*	
2nd		81		40.30		113		50.45			
3rd		93		46.27		33		14.73			
*Religion*											
Hindu		161		80.10		197		87.95		0.271	
Muslim		28		13.93		21		9.38			
Christian		12		5.97		6		2.68			
*Caste*											
General		69		34.33		91		40.63		0.927	
OBC		75		37.31		65		29.02			
SC		35		17.41		46		20.54			
ST		22		10.95		22		9.82			
*Socioeconomic class*											
Upper class		4		1.99		11		4.91		0.001*	
Middle class		39		19.40		112		50.00			
Lower class		158		78.61		101		45.09			
*Duration of giving breastfeeding (months)*											
0-12		31		15.42		120		53.57		0.001*	
13-24		27		13.43		49		21.88			
>24		143		71.14		55		24.55			
No		136		39.42		209		60.58			
*Child brushes teeth under parent supervision*											
Yes		33		16.42		128		57.14		0.001*	
No		168		83.58		96		42.86			

*Signifies that the p-value is less than 0.05 and is statistically significant

**Table 4:** **Table 4:** Logistic regression model determining risk factors for dental caries among preschool children (N = 425)

*Characteristics*		*OR (95% CI)*		*AOR (95% CI)*		*p-value*	
*Age of the children (months)*							
36-48		Ref		Ref		Ref	
49-60		3.62 (2.21-5.92)		2.53 (1.41-4.52)		0.002*	
61-72		10.01 (5.89-17.34)		5.39 (2.72-10.67)		0.001*	
*Gender*							
Male		Ref		Ref		Ref	
Female		0.82 (0.56-1.21)		1.15 (0.68-1.93)		0.596	
*Religion*							
Hindu		Ref		Ref		Ref	
Muslim		1.63 (0.89-2.98)		0.90 (0.36-2.29)		0.839	
Christian		2.44 (0.89-6.66)		1.97 (0.38-10.20)		0.416	
*Caste*							
General		Ref		Ref		Ref	
OBC		1.52 (0.96-2.40)		1.08 (0.55-2.11)		0.820	
SC		1.00 (0.58-0.72)		0.85 (0.42-1.74)		0.671	
ST		1.31 (0.67-2.57)		0.71 (0.24-2.10)		0.547	
*Child birth order in a family*							
1st		Ref		Ref		Ref	
2nd		2.07 (1.22-3.49)		1.36 (0.71-2.58)		0.347	
3rd		8.14 (4.50-14.70)		2.91 (1.38-6.14)		0.005*	
*Socioeconomic class*							
Upper class		Ref		Ref		Ref	
Middle class		0.95 (0.28-0.3.18)		0.79 (0.19-3.29)		0.876	
Lower class		4.30 (1.33-13.87)		2.19 (0.54-8.79)		0.269	
*Duration of giving breastfeeding (months)*							
0-12		Ref		Ref		Ref	
13-24 months		2.13 (1.1547-3.94)		2.13 (1.04-4.36)		0.037*	
>24 months		10.06 (6.08-16.63)		5.41 (2.97-9.85)		0.001*	
*Children brushes their teeth under any supervision*							
Yes		Ref		Ref		Ref	
No		6.78 (4.29-10.72)		2.70 (1.55-4.69)		0.001*	

*Signifies that the p-value is less than 0.05 and is statistically significant

### Sociodemographics and Dental Caries

This study was unable to show the relationship between gender and dental caries. A similar type of study in Jammu & Kashmir showed that gender had no relationship with dental caries.^22^ However, in some other studies, it had been shown that male child was one of the predictors of dental caries, boys were more inclined to develop dental caries as compared with the girl child, as it may be due to males being given precedence in Indian perspective^20^ and in some studies, girl children were more on verge of producing dental caries as compared with the boys because girls have an early flare-up of teeth as compared with the boys, which results in longer time contact with the oral environmental factors which leads to dental caries.^23^ In contrast to other countries, in Sri Lanka, girl child was more prone to dental caries as compared with the male child^2^ and in some studies, it showed that gender had no significant difference with dental caries among preschool children.^19^ This study showed that increasing age of the children was related to dental caries. A similar type of study conducted in Jammu and Kashmir^22^ and Bangalore^11^ showed that increase in age of the children was associated with dental caries. The possible reason could be that the standard of living of the parents and children changes with the evolution of years.^24^ This study was able to show that more than two children were associated with dental caries among preschool children. A similar type of study done in Sri Lanka showed that more than two children had a relationship with dental caries among preschool children.^25^ The probable reason could be that the parents distribute their care equally to all of their children the importance of oral hygiene; the care they can provide to a single child, they could not provide the same level of oral hygiene to their subsequent babies.^26^ The socioeconomic status of a family plays a very important role in developing dental caries among preschool children. However, this study failed to show the relationship between socioeconomic status and dental caries. A similar type of study conducted in Sudan showed a similar result that the socioeconomic status plays no role in dental caries.^19^ However, a study done in Brazil shows that low socioeconomic status had a positive relationship with dental caries and the probable reason could be the reluctance to reward the dental services, and poor awareness about the good oral hygiene practices.^26^

### Breastfeeding and Dental Caries

This study depicted that increasing duration of breastfeeding was found to be a significant risk factor for developing dental caries among the young growing children. A similar type of study was conducted in Brazil^27^ and Southwestern Nigeria^28^ where they depicted that increasing duration of the breastfeeding had a positive relationship with dental caries among preschool children. One of the significant reviews suggested that mother’s milk contains the oligosaccharide which acts as a component for bacteria to grow and results in developing dental caries due to prolonged exposure to breastfeeding.^29^

### Oral Hygiene Practice and Dental Caries

This study was able to show that children who did not brush the teeth under the supervision of the parents had a strong positive relationship with developing of dental caries. A similar type of result was shown in Bangalore about children who did not brush the teeth under supervision and who were more to develop dental caries as compared with the children who brushed the teeth under the supervision of parents.^11^ One of the dissimilar studies found that brushing under the supervision of the parents had no association with dental caries.^24^

### Methodological Consideration

This study used the random sampling technique, which means that every accomplice had an equal opportunity to fall into this study. The multivariable logistic regression model was computed for controlling the confounders. Apart from the strength, the study finds some limitations; since it was a cross-sectional in nature, a contributing temporality cannot be recognized between the exposure and the outcome.

## CONCLUSION

This study depicted that the increase in age, increased number of children, longer duration of breastfeeding, and the children who did not brush the teeth under the parent’s supervision were more vulnerable to develop dental caries. These are some of the risk factors obligatory to evaluate in future, such as the microbiological colony level of the *S. mutans* and hereditary history of the parents. The oral health education should be given at the grassroots level in AWCs where it is generally regarded as the education hub for the financially backward preschool children and this can be set as a platform for giving oral health education to preschool children and their mothers or caretakers. There is also a need for giving behavioral change training to the parents for changing the concept of longer duration of breastfeeding and oral hygiene practices so that it can help to reduce the burden of dental caries among the preschool age group which is the most vital period for the maturation of the teeth.
